# Optimization of Ultrasonic-Enzymatic-Assisted Extraction of Flavonoids from Sea Buckthorn (*Hippophae rhamnoides* L.) Pomace: Chemical Composition and Biological Activities

**DOI:** 10.3390/foods14101656

**Published:** 2025-05-08

**Authors:** Wenyu Suo, Wenzhe Wang, Dajing Li, Haihong Wu, Haiyan Liu, Wuyang Huang, Yanhong Ma

**Affiliations:** 1 School of Food and Biological Engineering, Jiangsu University, Zhenjiang 212013, China; 13673791093@163.com; 2 Institute of Agro-Product Processing, Jiangsu Academy of Agricultural Sciences, Nanjing 210014, China; 16611619825@163.com (W.W.); lidajing@163.com (D.L.); 20040019@jaas.ac.cn (H.W.); 3 The Work of Forestry Administrative Station of Kirgiz Autonomous Prefecture, Artush 845350, China; liuhaiyan02@caas.cn

**Keywords:** *Hippophae rhamnoides* L., flavonoids, ultrasonic-enzymatic-assisted extraction, antioxidant, α-glucosidase and α-amylase inhibition

## Abstract

Sea buckthorn pomace (SBP) is a rich source of flavonoid compounds with potential healthy properties. This study optimized ultrasonic-enzymatic-assisted extraction (UEAE) of flavonoids from SBP and investigated its chemical composition and biological activities. Under the optimal conditions (pectinase addition of 1500 U/g, ultrasonic power of 300 W, ethanol concentration of 48%, liquid–solid ratio of 34:1, extract temperature of 50 °C, and extraction time of 28 min), the yield of SBP flavonoid extracts (SBFEs) was 21.57 ± 0.45 mg/g, well-matched with the predicted value (21.72 mg/g). The chemical composition was detected by ultrahigh-performance liquid chromatography with quadrupole time-of-flight mass spectrometry (UPLC-QTOF-MS^E^) and mainly including isorhamnetin, kaempferol, and quercetin’s derivatives. After purification with AB-8 macroporous resin, the purified product (PSBFE) exhibited a significantly enhanced scavenging capability for 1,1-diphenyl-2-picryl-hydrazyl radical (DPPH) and 2,2′-azino-bis(3-ethylbenzothiazoline-6-sulfonic acid) (ABTS) (947.17 ± 3.85 and 427.33 ± 0.67 μmol Trolox/g, respectively) and ferric reducing antioxidant power (2.68 ± 0.01 mmol FeSO_4_·7H_2_O/g). Moreover, PSBFE possessed a pronounced inhibitory rate on α-glucosidase and α-amylase, with the IC_50_ at 52.89 ± 0.09 and 97.81 ± 0.42 μg/mL, respectively. These findings indicate that it is a reliable, optimal extraction method to obtain potential antioxidant and hypoglycemic flavonoids from SBP for comprehensive development in functional food.

## 1. Introduction

Sea buckthorn (*Hippophae rhamnoides* L.), a perennial shrub belonging to the family Elaeagnaceae, is widely cultivated in Asia, Europe, and North America [1]. There are 7 species and 11 subspecies of *Hippophae* L. in the world. Moreover, seven species and seven subspecies of *Hippophae* L. are distributed in China, with a total area of about 2.7 million hm^2^, accounting for more than 90% of the growth area of sea buckthorn in the world [2]. With its outstanding tolerance to drought, wind erosion, and sand burial, sea buckthorn exhibits extraordinary survival capabilities in saline-alkali environments. These adaptive traits have established it as a premier species for erosion control and land rehabilitation, contributing substantial ecological benefits to fragile ecosystems [3]. As a dual-purpose plant with medicinal and nutritional applications, *Hippophae rhamnoides* L. (sea buckthorn) has been recorded in historical Chinese medical literature dating back to the Tang and Ming dynasties, with detailed descriptions of its various pharmacological effects [4]. Sea buckthorn is popular for its bright orange and delicious berries, which are rich in bioactive compounds such as vitamins (C, E, and K), flavonoids, carotenoids, and omega fatty acids [5]. Traditionally used in medicine and nutrition [5], sea buckthorn berries are valued for their antioxidant, anti-inflammatory, and immune-boosting properties [6]. The berries are processed into various products, including juices, oils, and dietary supplements. During juice extraction, a significant byproduct called sea buckthorn pomace (SBP) is generated. This pomace, consisting of seeds, skins, and pulp residues, retains a substantial amount of nutrients and bioactive compounds, such as flavonoids and dietary fibers [7]. Qin et al. reported that dietary SBP supplementation could enhance muscle mass, improve meat tenderness, increase water-holding capacity, and boost antioxidative properties [8]. Thus, SBP represents a sustainable resource with significant potential for utilization in the food, pharmaceutical, and cosmetic industries, contributing to both economic and environmental benefits.

Flavonoids, abundantly present in sea buckthorn fruits, branches, and seeds, constitute principal bioactive components with various health benefits, including attenuating organ senescence, mitigating cardiovascular diseases, and modulating glycemic control [9]. Wang et al. systematically investigated the antioxidant and neuroprotective mechanisms of total sea buckthorn flavonoids [10]. Their findings revealed that sea buckthorn flavonoids exhibit potent scavenging capacities against the DPPH radical, hydroxyl radical, and superoxide anion, thereby confirming their dual role as primary antioxidants and neuroprotective agents. Further validation through experiments utilizing a *Hidradenitis elegans* mutant model corroborated the neuroprotective efficacy of sea buckthorn flavonoids in vivo [10]. Notably, even the processing byproduct, SBP, retains a significant flavonoid content. Hao et al. explored the therapeutic potential of flavonoids derived from SBP in obesity management, benchmarking their effects against the conventional anti-obesity drug rosiglitazone [11]. The study confirmed that SBP flavonoids possess anti-obesity properties and hepato-protective effects. Therefore, the comprehensive utilization of SBP through advanced extraction technologies is meaningful and valuable.

Conventional flavonoid extraction techniques, including organic solvent extraction (SE) and heat reflux (HF), are constrained by inherent limitations, such as prolonged operational durations, thermal degradation of thermolabile flavonoid structures, and low extraction efficiency [12]. Emerging green extraction technologies, notably ultrasonic-assisted extraction (UAE), microwave-assisted extraction (MAE), and enzymatic-assisted extraction (EAE), have recently demonstrated superior efficacy in extracting total flavonoids with enhanced preservation of bioactive integrity [13,14]. These advanced methodologies exhibit distinct advantages over traditional approaches, including reduced energy consumption and improved environmental friendliness, meaning they have gained popularity [15]. Critically, hybrid extraction strategies that synergistically combine multiple techniques (e.g., UAE coupled with EAE) have emerged as a paradigm-shifting solution. Such integrations capitalize on complementary mechanisms—for instance, ultrasonic cavitation-induced cell wall disruption and enzymatic hydrolysis of polysaccharide matrices—to amplify extraction efficiency while mitigating individual limitations like incomplete matrix penetration in EAE or localized overheating in UAE [16].

This study proposes an effective approach, ultrasonic-enzymatic-assisted extraction (UEAE), to optimize total flavonoid recovery from SBP, including systematic parameter optimization via single-factor experiments and a response surface methodology, purification of crude extracts using AB-8 macroporous adsorption resin, and a comparative evaluation of the antioxidant capacity and hypoglycemic properties of SBP flavonoids. This multistage investigation provides critical insights for advancing the valorization of agricultural byproduct SBP through sustainable extraction engineering.

## 2. Materials and Methods

### 2.1. Plant Material

Xinjiang Zhongke Sea Buckthorn Technology Co., Ltd. (Kezhou, China) kindly provided the sea buckthorn pomace, which was a processing byproduct derived from fresh fruits of the cultivated variety *Hippophae rhamnoides* L. “Shenqiuhong” after physical pressing into juice. The pomace was obtained through solid–liquid separation after juice extraction, followed by low-temperature drying. No enzymes were used during the pressing process. Following the removal of seeds, the pomace was ground into a fine powder, and stored at 4 °C for further experiments within six months.

### 2.2. Chemicals

Ethanol, *L*-Ascorbic acid (vitamin C, VC), cellulose (10,000 U/g), and pectinase (30,000 U/g) were sourced from Shanghai Macklin Biochemical Technology Co., Ltd. (Shanghai, China). Macroporous resin AB-8 was obtained from Shanghai Lanji Technology Development Co., Ltd. (Shanghai, China). Acarbose, 3,5-dinitrosalicylic acid (DNS), soluble starch solution, p-nitrophenyl-α-D-glucopyranoside (pNPG), α-glucosidase, α-amylase, DPPH, ABTS, 6-hydroxy-2,5,7,8-tetramethylchroman-2-carboxylic acid (Trolox), and 2,4,6-tri(2-pyridyl)-s-triazine (TPTZ) were purchased from Shanghai Yuanye Bio-Technology Co., Ltd. (Shanghai, China). All solvents and reagents utilized in this study were of analytical grade.

### 2.3. Extraction Procedure of SBP

We precisely weighed and extracted 1.000 g of SBP using an XM-300UVF Ultrasonic Cleaner (Xiaomei Ultrasound Instrument (Kunshan) Co., Ltd., Kunshan, China). In preliminary experiments, the impacts of the following extraction method on flavonoid yield from sea buckthorn pomace were evaluated: (1) solvent extraction (SE, 50% ethanol solution alone was used, no ultrasound), (2) ultrasonic-assisted extraction (UAE, 300 W ultrasonic power), (3) enzymatic-assisted extraction (EAE, 1500 U/g pectinase addition), and (4) ultrasonic-enzymatic-assisted extraction (UEAE, ultrasound combined with pectinase addition). All extraction was conducted at 50 °C for 30 min with a liquid–solid ratio of 1:25. The results indicated that UEAE was the most effective method. Consequently, UEAE was chosen for subsequent single-factor experiments.

### 2.4. Single-Factor Experiments

The effects of various factors on total flavonoid yield were examined by single-factor experiments. The single factors included the enzyme ratio (pectinase: cellulase = 0:1, 1:1, 1:2, 2:1, and 1:0), enzyme addition (1000, 1500, 2000, 2500, and 3000 U/g), extraction temperature (20, 30, 40, 50, and 60 °C), extraction time (10, 20, 30, 40, and 50 min), ultrasonic power (0, 100, 150, 200, 250, and 300 W), ethanol concentration (40%, 50%, 60%, 70%, 80%, and 90%) and liquid–solid ratio (10:1, 15:1, 20:1, 25:1, 30:1, and 35:1 mL/g). In each experimental group, only one variable was altered while maintaining all other parameters at predetermined baseline values (extraction temperature: 50 °C; enzyme addition: 1500 U/g; liquid–solid ratio: 1:25; ethanol concentration: 50%; ultrasonic power: 300 W; extraction time: 30 min) to systematically evaluate the influence of individual variables on the target response.

### 2.5. Box–Behnken Design and Analysis

Based on the preliminary results of the single-factor test, a three-level, three-factor BBD in Design Expert 13 software (StatEase^®^, Minneapolis, MN, USA) with the response surface methodology (RSM) was conducted to optimize the flavonoid extraction yield in SBP. This powerful mathematical and statistical technique is useful for finding the relationship between the independent variable factors and the response to optimize the conditions. As shown in Table S1, independent variables included the extraction time (A: 15, 30, and 45 min), the ethanol concentration (B: 40, 60, and 80%), and the liquid–material ratio (C: 20:1, 30:1, and 40:1). Based on the results of previous single-factor experiments, the flavonoid extraction yield was taken as the assessment index of each independent variable. This study involved 17 experimental runs, including 5 replicates at the central point, which were utilized to determine the experimental error and data reproducibility [17]. To minimize the impact of potential experimental variability, all trials were conducted in a randomized order with triplicate measurements for each condition.

### 2.6. Purification Procedure of SBP

The obtained extracts underwent centrifugation at 5000× *g* for 10 min at room temperature, and the supernatant obtained was considered the crude sea buckthorn flavonoid extract (SBFE). The crude SBFE obtained using the optimal extraction conditions was further purified by macroporous resin AB-8. It was dissolved in deionized water (final concentration: 10 mg/mL) and mixed with activated AB-8 resin at a 1:50 (resin concentration: 20 mg/mL) ratio for static adsorption purification. Then, it was concentrated with a rotary evaporator at 50 °C and vacuum of 0.1 MPa. Next, after 12 h of pre-freezing at −80 °C, the sample was freeze-dried at 10 Pa, and the cold trap was operated at −50 °C. The final purified product (PSBFE) was obtained for subsequent comparison.

### 2.7. Determination of Total Flavonoid Content (TFC)

Total flavonoid content (TFC) was determined by modified sodium nitrite–aluminum nitrate colorimetry [18]. Briefly, an extract solution (2.0 mL) was prepared in a 10 mL tube. Subsequently, solutions of 5% sodium nitrite (NaNO_2_, 0.2 mL), 10% aluminum nitrate (Al(NO_3_)_3_, 0.2 mL), and 4% sodium hydroxide (NaOH, 2 mL) were added at 5 min, 5 min, and 15 min intervals. The absorbance value at 510 nm was recorded on an Epoch Microplate Spectrophotometer (Boweigaoke Biotechnology Science and Technology Co., Ltd., Beijing, China). The rutin standard curve was linear at 510 nm (*Y* = 0.0025 *X* + 0.0391, *R*^2^ = 0.9997). The TFC was calculated according to the standard curve linear at 510 nm, and flavonoid extraction yield was expressed as milligrams of rutin equivalents per gram of SBP dry weight (mg/g).

### 2.8. UPLC-QTOF-MS Analysis

Chromatographic peaks of PSBFE were identified and confirmed through qualitative analysis using UPLC-QTOF-MS^E^, which consisted of an accurate Q-TOF mass spectrometer (Agilent 6538 UHD, Agilent Technologies, Santa Clara, CA, USA) equipped with an electrospray ionization (ESI) interface coupled with an Agilent 1290 Infinity UHPLC system (Agilent Technologies, Santa Clara, CA, USA). Chromatographic separation was performed on a Waters XSelect HSST3 (2.1 × 100 mm, 2.5 μm) column using 0.1% formic acid in water (phase A) and acetonitrile (phase B) as mobile phases. The linear gradient program was 0–5 min, 14% B; 5–10 min, 14–18% B; 10–16 min, 18–20% B; 16–20 min, 20–28% B; 20–25 min, 28–32% B; and 25–35 min, 32–45% B, at a flow rate of 0.4 mL/min with an injection volume of 3 μL. The mass spectrometry scanning was performed with ESI as the ionization source in both positive and negative modes for comprehensive qualitative analysis. The mass spectrometer parameters were optimized as follows: capillary voltage 4000 V (positive) and 3500 V (negative), gas temperature 350 °C, dry gas flow rate 11 L/min, nebulizer gas pressure 45 psi, fragmentor voltage 120 V, skimmer voltage 50 V (positive) and 45 V (negative), and Octopole RF Peak 750 V. MS/MS collision energies were set to 10 V, 20 V, and 40 V, respectively, to obtain fragmentation patterns for structural elucidation. ESI was performed with the scan range between *m*/*z* 100 and 1000. For compound identification in this qualitative study, the acquired high-resolution mass spectra were matched against the SCIEX-provided TML TCM database using the following criteria: mass error < 10 ppm, isotope pattern difference < 10%, XIC (extracted ion chromatogram) intensity > 100 cps, and signal-to-noise ratio (S/N) > 3. The data acquisition workstation was Mass Hunter Acquisition, and the offline analysis software was used for comprehensive qualitative analysis. System suitability was verified by analyzing reference standards to ensure mass accuracy (<2 ppm) and retention time stability throughout the analytical sequence.

### 2.9. In Vitro Antioxidant Capacity

#### 2.9.1. DPPH Assay

DPPH radical scavenging activity was determined based on the reduction in DPPH radical, according to a previous report and with slight modifications [19]. Briefly, 2 mL of 6 × 10^−5^ M methanolic solution of DPPH (2,2-diphenyl-1-picrylhydrazyl) was added to 200 µL of sample and incubated for 15 min in the dark. Then, the absorbance was measured at 517 nm. Antioxidant activity was calculated using a calibration curve obtained with Trolox. The results were expressed in μmol Trolox/g sample.

#### 2.9.2. ABTS Assay

For the ABTS assay, the procedure followed the method of Thaipong et al. with some modifications [19]. The stock solutions included 7 mM ABTS radical·+ solution and 2.45 mM potassium persulfate solution. The working solution was then prepared by mixing the two stock solutions in equal quantities and allowing them to react for 16 h at room temperature in the dark. Thereafter, the solution was diluted with phosphate buffer (PBS, pH 7.4) to an absorbance of 0.700 ± 0.05 at 734 nm. The decrease in absorbance was read at 6 min after the addition of 2.0 mL of diluted ABTS·+ solution to 20 µL of samples. A standard curve was generated by replacing the sample with Trolox at different concentrations. The results were expressed in μmol Trolox/g sample.

#### 2.9.3. Ferric Reducing Antioxidant Potential (FRAP) Assay

For the FRAP assay, the procedure followed the method of Thaipong et al. with some modifications [19]. The FRAP reagent was prepared with 200 mmol/L acetate buffer (pH 3.6), 10 mmol/L of 2,4,6-tripyridyl-s-triazine (TPTZ) solution in 40 mmol/L HCl, and 20 mmol/L FeCl_3_·6H_2_O in distilled water in the proportions of 10:1:1, respectively. The samples (100 µL) were mixed with the FRAP reagent (3 mL), and the absorbance was measured at 593 nm after 6 min. FeSO_4_·7H_2_O was used as the standard, and the antioxidant activities are expressed as mmol FeSO_4_·7H_2_O/g sample.

### 2.10. In Vitro Enzyme Inhibitory Effect

#### 2.10.1. α-Amylase Inhibition Assay

The α-amylase inhibitory effect was determined according to a previous report [20]. The sample of SBFE, PSBFE, and acarbose solutions were prepared in phosphate-buffered saline (PBS) to different concentration gradients. In the first step, 50 µL of sample was added to 50 µL of α-amylase solution (0.1 mg/mL) and incubated at 37 °C for 10 min, respectively. Then, 100 µL soluble starch solution (1%) was added and incubated at 37 °C for 10 min. Finally, 400 µL DNS was added and maintained at 100 °C for 10 min. The absorbance was measured at 540 nm. Phosphate-buffered saline was used as a sample negative control and control blank, and acarbose was used as a positive control. The inhibition rate of α-amylase was calculated following Equation (1)

(1) $$\mathrm{Inhibition}\;\mathrm{percentage}\;(\%)=\left(1-\frac{A_{1}-A_{2}}{A_{3}-A_{4}}\right)\times 100\%$$

where *A*_1_: the absorbance of sample reaction, *A*_2_: the absorbance of sample negative control, *A*_3_: the absorbance of control reaction, *A*_4_: the absorbance of control blank.

#### 2.10.2. α-Glucosidase Inhibition Assay

The procedure of the α-glucosidase inhibition assay was similar to that of α-amylase [20]. This used α-glucosidase instead of α-amylase, while p-nitro-phenyl-α-D-glucopyranoside (pNPG, 6 mM) was used instead of starch and reacted for 30 min. Finally, 500 μL of Na_2_CO_3_ solution was added and determined at 400 nm. The inhibition rate of α-glucosidase was calculated by the same formula as Equation (1).

### 2.11. Statistical Analysis

Each analysis was performed in triplicate, and the data are reported as mean values ± standard deviation (SD). Response surface analysis was performed by Design Expert version 13. Databases and statistical analyses were performed by GraphPad Prism 9.5 (GraphPad Software, Inc., La Jolla, CA, USA). One-way analysis of variance (ANOVA) was used for multiple comparisons by GraphPad Prism 9.5 software followed by Tukey’s multiple comparisons test. The difference was considered to be statistically significant if *p* < 0.05.

## 3. Results and Discussion

### 3.1. Single-Factor Experimental Analysis of UEAE

A pre-experiment compared SE, UAE, EAE, and UEAE. Either ultrasonic or enzymatic extraction was superior to direct ethanol extraction, and a synergistic effect existed between ultrasonication and enzymolysis (Figure S1, Table S2). Under the influence of ultrasound, solvent molecules rapidly enter a vibrational state and are further accelerated through energy transfer, thereby enhancing enzyme–substrate interactions and improving the efficiency of enzymatic hydrolysis. Additionally, the elevated temperature of the reaction system promotes molecular vibration, amplifying both the thermal effects of ultrasound and the synergistic dynamics of the reaction [21]. The unique penetrating power of ultrasound, as well as the hydrolysis ability of cellulose or pectinase, can destroy the cell walls of plant cells and help to release intracellular active substances, resulting in higher extraction. Thus, UEAE was further explored for the optimal conditions by single-factor experiments.

#### 3.1.1. Effect of Enzyme Ratio on the Flavonoid Extraction Yield

**Figure 1:**
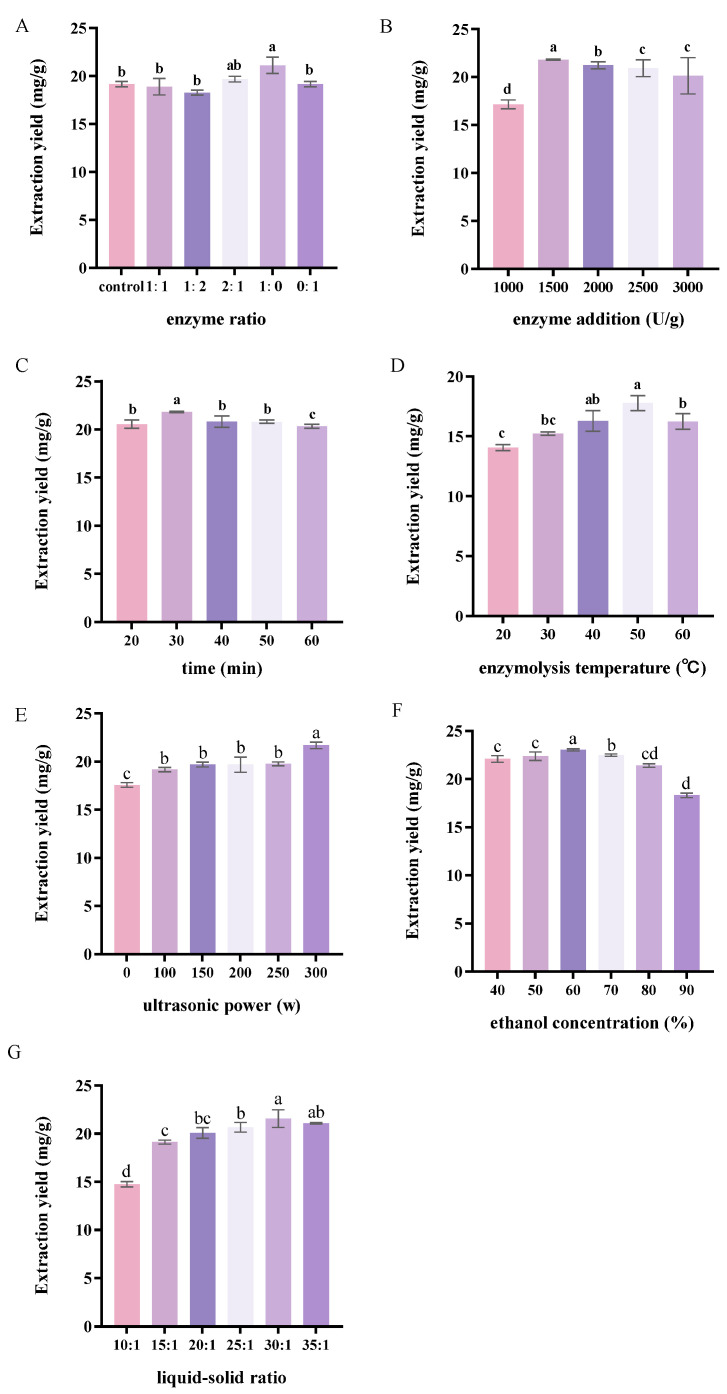
Effect of various factors on flavonoid extraction yield from SBP. (**A**) The effect of enzyme ratio. (**B**) The effect of enzyme addition (U/g). (**C**) The effect of extraction time. (**D**) The effect of extraction temperature (°C). (**E**) The effect of ultrasonic power (W). (**F**) The effect of ethanol concentration (%). (**G**) The effect of liquid–solid ratio. Different letters indicated statistically significant differences between different treatments (Tukey, *p* < 0.05).

Figure 1A shows the impact of the enzyme ratio on the SBFE. The addition of pectinase significantly increased the extraction yield of flavonoids and reached a maximum value of 21.11 ± 0.85 mg/g, whereas the addition of cellulase did not significantly affect the flavonoid extraction yield. The combined enzymatic hydrolysis using pectinase and cellulase was not as effective as single pectinase. This may be due to the specific activities of different enzymes, which target different components of the plant cell wall and promote the release of substances of different complexity [22]. Yu-Jie et al. also found pectinases more effective for the extraction of flavonoids, such as luteolin and apigenin. Pectinases encompass a group of enzymes that collectively exhibit three main types of activities, including pectin esterase, polygalacturonase, and pectin lyase. Together, these enzymes work synergistically to degrade pectin, facilitating the breakdown of the cell wall and the extraction of bioactive compounds like flavonoids [23]. Sea buckthorn flavonoid fractions were more sensitive to pectinase here. Consequently, pectinase was selected for the extraction of SBP.

#### 3.1.2. Effect of Enzyme Dosage on the Flavonoid Extraction Yield

As illustrated in Figure 1B, the yield of SBFE was significantly influenced by the dosage of enzyme added. When the pectinase addition increased from 1000 to 1500 U/g, the yield of SBFE increased and reached its maximum value of 21.83 ± 0.07 mg/g. Afterwards, the extraction yield gradually decreased with an increasing enzyme dosage. Higher levels of enzymatic supplementation may enhance the interaction between pectinase and the cellular structure of SBP cell walls, increase membrane permeability, and accelerate diffusion rates, thereby improving the efficiency of flavonoid extraction [24]. However, excessive enzyme addition may lead to oversaturation, as the high enzyme concentration can cause aggregation, preventing some enzyme molecules from effectively interacting with SBP cells [25]. Consequently, the optimal enzyme dosage for flavonoid extraction was determined to be 1500 U/g.

#### 3.1.3. Effect of Extraction Time on the Flavonoid Extraction Yield

The effect of extraction time on the yield of SBFE is illustrated in Figure 1C. As the extraction time increased, the flavonoid extraction yield initially rose, peaking at 30 min with a maximum value of 21.76 ± 0.06 mg/g, after which it gradually declined. A sufficient extraction time ensures thorough interaction between the enzyme and substrate, as well as optimal utilization of ultrasonic energy. During the initial phase, ultrasound waves disperse into the extraction medium, generating cavitation that produces thermal and mechanical effects, thereby enhancing the extraction efficiency [26]. However, prolonged ultrasound exposure can create high-temperature and high-pressure conditions due to excessive cavitation, potentially degrading the flavonoids [21]. Additionally, extended extraction times may lead to the dissolution of competing substances in the limited solvent system, which can inhibit flavonoid solubility [27]. In the anthocyanin extraction experiments, there was no significant increase or even a decrease in the extraction rate of the 60 min extraction group compared with the 30 min extraction group, which could be attributed to the prolonged ultrasonic exposure resulting in mechanical shear stress or chemical oxidation, leading to structural degradation of the active components [28]. Previous studies have shown that the optimal time for UAE of phenolic compounds from plant byproducts is 10–90 min, beyond which irreversible degradation of the target components may occur [29]. Thus, the optimal extraction time for flavonoid extraction was determined to be 30 min.

#### 3.1.4. Effect of Extraction Temperature on the Flavonoid Extraction Yield

When the extraction temperature ranged from 20 to 60 °C, the yield of SBFE initially increased and then decreased, reaching its peak of 17.78 ± 0.86 mg/g at 50 °C (Figure 1D). As bioactive molecules, enzymes exhibit high sensitivity to temperature variations, which significantly influence their catalytic activity and spatial conformation [30]. As the temperature approaches the enzyme’s optimal working range, its catalytic efficiency gradually increases, reaching maximum activity. However, exceeding this critical threshold can lead to denaturation and deactivation of the enzyme protein, as well as potential alterations in the molecular structure of flavonoid compounds, ultimately reducing extraction efficiency. Temperature elevation enhances solute desorption capacity and solubility in the solvent while simultaneously reducing the solvent viscosity, thereby improving its diffusivity within the tissue matrix [29]. Beyond optimal temperatures, the extraction efficiency declines owing to the attenuation of cavitation phenomena [31]. Thus, the optimal extraction temperature for flavonoid extraction was determined to be 50 °C.

#### 3.1.5. Effect of Ultrasonic Power on the Flavonoid Extraction Yield

The impact of ultrasonic power on the yield of SBFE is illustrated in Figure 1E. As the ultrasonic power increased, the extraction yield rose until it reached a maximum value of 21.96 ± 0.33 mg/g at 300 W. The intensity and duration of ultrasonic treatment are critical parameters that significantly affect the extraction efficiency of bioactive compounds, with a 300 W power setting demonstrating a superior performance in promoting flavonoid dissolution [32]. Higher power levels generate more pronounced cavitation effects, accelerating the release and migration of flavonoid compounds from SBP cells, and thereby increasing flavonoid extraction [33]. However, excessively high ultrasonic power can lead to the formation of oversized cavitation bubbles that fail to collapse or experience a significant reduction in collapse frequency, ultimately impairing the extraction efficiency of target components [34]. Therefore, the optimal ultrasonic power for flavonoid extraction was determined to be 300 W.

#### 3.1.6. Effect of Ethanol Concentration on the Flavonoid Extraction Yield

As shown in Figure 1F, when the ethanol concentration increased from 40% to 60%, the yield of SBFE gradually rose to a maximum of 23.05 ± 0.11 mg/g. The ethanol concentration significantly affects the polyphenol extraction efficiency, and higher ethanol concentrations lead to increased flavonoid extraction yields, consistent with previous studies [35]. However, further increases in ethanol concentration led to a decline in yield. This trend may be primarily due to the relationship between solvent polarity, the chemical structure of flavonoids, and their solubility properties. Ethanol disrupts hydrogen bonds and hydrophobic interactions between flavonoids and macromolecules such as proteins and polysaccharides in plant cell walls, facilitating the release of flavonoids [36], while water is a highly polar solvent that effectively dissolves polar flavonoid glycosides [27]. Optimal ethanol–water mixtures demonstrate a superior extraction performance compared to pure solvents. The synergistic effect occurs through dual mechanisms: water induces cell wall expansion to enhance solvent penetration, while ethanol facilitates polyphenol dissolution. This binary system creates an ideal polarity environment by modifying dielectric properties, enabling efficient extraction of both polar and non-polar polyphenols [37]. Flavonoids typically exist in two forms, flavonoid glycosides and aglycones, which have different polarities. Moderate ethanol concentrations (e.g., 60%) provide a balanced polarity that can effectively dissolve both polar and moderately polar flavonoids [27]. Therefore, the optimal ethanol concentration for flavonoid extraction was determined to be 60%.

#### 3.1.7. Effect of Liquid–Solid Ratio on the Flavonoid Extraction Yield

The liquid–solid ratio is a critical extraction parameter influencing the yield of SBFE. As shown in Figure 1G, the yield of SBFE increased with the liquid–solid ratio, reaching a peak of 21.57 ± 0.92 mg/g at a ratio of 30:1 mL/g, after which it began to decline. As the ratio increases, more solvent penetrates the plant tissue cells, facilitating the release and diffusion of flavonoids into the solvent, thereby increasing the extraction yield [38]. However, beyond a certain point, further increases in the liquid–solid ratio may lead to saturation of the active ingredients, resulting in solvent wastage and the leaching of impurities [39]. Conversely, if the liquid–solid ratio is too low, the extraction of flavonoids from the raw material may be incomplete. Therefore, the optimal liquid–solid ratio for flavonoid extraction was determined to be 30:1 mL/g.

### 3.2. Response Surface Optimization of UEAE

#### 3.2.1. Statistical Analysis and Model Fitting

The data in Table 1 were analyzed using a quadratic response surface regression model, and a quadratic multiple regression equation for flavonoid yield was obtained:

*Y *= 0.4454*A* − 1.23*B* + 1.38*C* + 0.1365*AB* − 0.1862*AC* + 0.6170*BC* + 0.0085*A*^2^ − 0.9752*B*^2^ − 1.69*C*^2^ + 21.22

where *A*, *B*, and *C* were the coded values of extraction time, ethanol concentration, and liquid–solid ratio, respectively.

To validate the quadratic model, an *F*-test was performed and the analysis of variance (ANOVA) was ascertained, with the results summarized in Table S3. The model’s *p*-value < 0.0001 confirmed its significance and high suitability for the experiment. The *p*-values for the regression models were all below 0.05, indicating statistical significance and reliability for optimization. The lack-of-fit with a *p*-value at 0.8073 (*p* > 0.05) indicated that the loss was not significant, confirming the model’s adequacy [40]. The high correlation coefficient (*R*^2^ = 0.9794) indicated that the data reasonably reflect the flavonoid yield. The predicted *R*^2^ value (0.9092) was high, which was comparable to the adjusted *R*^2^ value (0.9529), conforming that the developed model was precise and credible. Thus, the model is reliable for optimizing flavonoid extraction from SBP using UEAE.

#### 3.2.2. Response Surface Analysis

**Figure 2:**
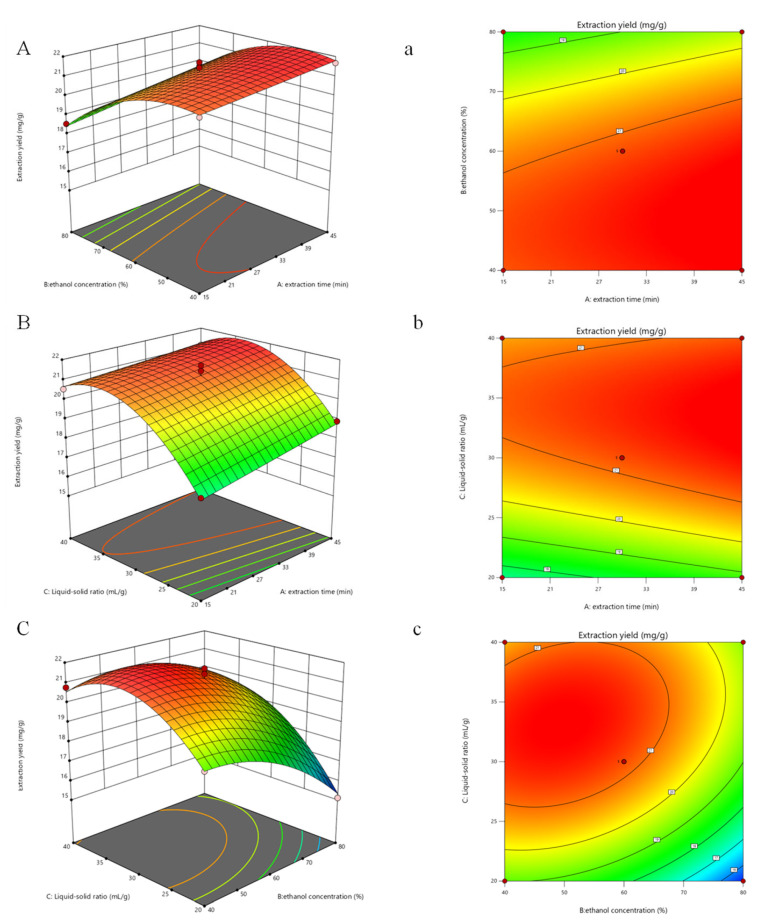
Three-dimensional response surface and contour plots of flavonoids extracted from sea buckthorn pomace: (**A**,**a**) extraction time and ethanol concentration; (**B**,**b**) extraction time and liquid–solid ratio; and (**C**,**c**) ethanol concentration and liquid–solid ratio.

The three-dimensional response surface visually demonstrates the sensitivity of the response value to variable changes, while the two-dimensional contour plot reveals the strength of interactions between variables [41]. An elliptical contour indicates a strong interaction, whereas a circular contour suggests a weak one. Furthermore, the steepness of the slope in the response surface graph reflects the sensitivity of the response value to influencing factors [42]. As illustrated in Figure 2, the slope between *BC* is relatively steep, and their interaction is significant (*p* < 0.05), whereas the slopes between *AB* and *AC* are relatively flat, indicating no significant interaction. Analysis of factor significance in Table S3 and response surface in Figure 2 both revealed that the liquid–solid ratio (*C*) had the greatest impact on flavonoid extraction, followed by ethanol concentration (*B*), and extraction time (*A*) had the least influence. Factors *B* and *C* had extremely significant synergies among them (*p* < 0.01), and quadratic terms (*B*^2^, *C*^2^) also significantly influenced extraction rates.

#### 3.2.3. Verification of the Extraction Process

Through response surface regression analysis, the optimal extraction conditions were determined as follows: extraction time of 28.01 min, liquid–solid ratio of 33.47:1, and ethanol concentration of 47.97%, with a predicted flavonoid yield of 21.72 mg/g. For practical application, these parameters were adjusted to an extraction time of 28 min, a liquid–solid ratio of 34:1, and an ethanol concentration of 48%. Validation experiments under these optimized conditions yielded an average flavonoid extraction of 21.57 ± 0.45 mg/g, closely aligning with the predicted value. This confirms the reliability and reproducibility of the optimized extraction process.

### 3.3. Preliminary Identification of Flavonoids in Sea Buckthorn Pomace

Flavonoids are characterized by a 15-carbon skeleton consisting of two benzene rings linked by an oxygen-containing heterocyclic pyran ring. Structural variations within this framework allow classification into subcategories such as flavones, flavonols, and flavanones [43]. These structural differences also confer distinct chromatographic behaviors and UV-visible spectral profiles, which are critical for their identification. Here, UPLC-QTOF-MS was employed to analyze flavonoid compounds in sea buckthorn pomace extract. Based on the precise molecular weight information provided by high-resolution mass spectrometry (HRMS), characteristic fragment ions generated by tandem mass spectrometry (MS/MS), retention time patterns in UPLC, elution-order characteristics during gradient elution, and characteristic absorption spectra obtained by ultraviolet–visible spectrophotometry (UV-Vis), combined with systematic comparison with relevant data from the published literature, this study successfully tentatively identified 14 flavonoid compounds, including subclasses such as flavonols and flavanones. Fourteen flavonoid constituents were preliminarily identified by matching molecular weights, fragmentation patterns, retention times, elution orders, and UV spectra with relevant references [44,45,46,47,48,49,50] (Table 2, the chromatogram at 360 nm shown in Figure S2). These included two flavanols: (-)-gallocatechin and *L*-epicatechin [45]; one condensed tannin: procyanidin B2 (a proanthocyanidin dimer) [46]; five flavonol glycosides: rutin (quercetin-3-*O*-rutinoside) [47], quercetin-3-*O*-glucoside [48], kaempferol-3-*O*-rutinoside [45], isorhamnetin-3-*O*-neohespeidoside [49], and kaempferol-3-*O*-glucoside [45]; four flavonols: myricetin [44], quercetin, kaempferol, and isorhamnetin [48]; one flavone glycoside: apigenin-7-glucoside [49]; and one flavanone: naringenin [50]. Among these, isorhamnetin-3-*O*-neohespeidoside exhibited the highest chromatographic peak intensity. The identified compounds are similar to previously reported flavonoid profiles of sea buckthorn fruits [44,45,46,47,48,49,50], confirming the predominance of isorhamnetin, kaempferol, quercetin, and their glycosylated derivatives in this species.

### 3.4. Antioxidant Activity

Antioxidants exert their effects through multiple mechanisms. Current in vitro methods for evaluating antioxidant capacity are based on the following principles: (1) direct scavenging of free radicals, (2) activation of antioxidant enzymes, (3) enhancement of metal chelation activity, and (4) inhibition of pro-oxidants, etc. [51]. Typically, two or more methods with distinct mechanisms are employed to assess the antioxidant activity of natural products. Commonly used assays include DPPH, ABTS, and FRAP. The DPPH radical, as a representative lipophilic free radical, is widely utilized for assessing antioxidant activity in lipid systems. Notably, this method specifically quantifies the specific scavenging efficiency of antioxidants toward the DPPH radical [43]. In contrast, ABTS generates a stable radical cation (ABTS^+•^) soluble in both aqueous and organic phases, which allows for simultaneous detection of hydrophilic and lipophilic antioxidants, providing a broader evaluation of total antioxidant capacity (TAC) [51]. However, the measured activity is strictly confined to the ABTS^+•^ system. The FRAP assay, on the other hand, is based on the reduction of ferric complexes to their ferrous form [43].

Flavonoids, rich in hydroxyl groups and featuring phenolic rings, hydroxyl side chains, and glycosylation modifications, exhibit strong free radical scavenging activity, making them highly effective antioxidants [51]. The crude extract (SBFE) demonstrated ABTS and DPPH scavenging capacities of 208.58 ± 2.21 μmol Trolox/g and 176.67 ± 0.51 μmol Trolox/g, respectively, along with a ferric ion reducing ability of 0.50 ± 0.01 mmol FeSO_4_·7H_2_O/g. After purification (PSBFE), these values significantly increased to 947.17 ± 3.85 μmol Trolox/g (ABTS), 427.33 ± 0.67 μmol Trolox/g (DPPH), and 2.68 ± 0.01 mmol FeSO_4_·7H_2_O/g (FRAP), as shown in Table 3. The enhanced antioxidant activity of the purified flavonoid extract was consistent with the findings of Jiang et al. [52]. Different flavonoids exhibit different antioxidant capacities, which probably depends upon the type, quantity, and arrangement of functional groups in the structure [43]. Tian et al. reported that kaempferol, apigenin, and quercetin had better ABTS radical scavenging activities than VC, among which quercetin had the strongest scavenging activity [53]. Quercetin and its derivatives (e.g., rutin) have been reported to be the most active antioxidant compounds in DPPH and FRAP assays of *Annona coriacea* extracts, whereas isorhamnetin derivatives possessed higher or similar antioxidant activity to quercetin derivatives in ABTS [54]. Apigenin-7-glucoside also possesses strong free radical scavenging properties [55]. The antioxidant capacity of PSBFE should be attributed to its main components, isorhamnetin, kaempferol, quercetin, and their derivatives, as well as apigenin-7-glucoside. These results indicate that flavonoids extracted from SBP possess antioxidant activity, highlighting their potential as a novel natural source for antioxidant development.

### 3.5. In Vitro Enzyme Inhibitory Potential

α-Amylase initiates carbohydrate digestion by hydrolyzing starch into oligosaccharides and disaccharides [56], which are subsequently cleaved into absorbable monosaccharides by α-glucosidase in the small intestine [51]. These monosaccharides enter the bloodstream, driving postprandial hyperglycemia—a critical concern in diabetes management. Thus, dual inhibition of α-amylase and α-glucosidase is critical for regulating carbohydrate digestion and mitigating hyperglycemia, and α-glucosidase inhibitors and α-amylase inhibitors have become effective therapeutic agents for controlling postprandial blood glucose elevation in clinical practice.

As shown in Table 4, both SBFE and PSBFE exhibited significant α-amylase inhibitory activity, suggesting that flavonoids in SBP can effectively suppress carbohydrate hydrolysis. The IC_50_ values for α-amylase inhibition were 316.70 ± 1.43 μg/mL (SBFE) and 97.81 ± 0.42 μg/mL (PSBFE), compared to 18.44 ± 0.08 μg/mL for the positive control acarbose. Notably, purification significantly enhanced inhibitory efficacy, as evidenced by the marked reduction in PSBFE’s IC_50_ value (*p* < 0.05). This result revealed that the flavonoids extracted from sea buckthorn pomace in this study exhibited significantly stronger α-amylase inhibitory activity than *Ligusticum chuanxiong* flavonoids (IC_50_ = 676.9 μg/mL) reported by Yao Wen et al. [57]. This superior performance underscores the potential of PSBFE as a novel candidate for hypoglycemic drug development, providing critical insights into plant-derived α-amylase inhibitors.

On the other hand, both SBFE and PSBFE exhibited α-glucosidase inhibitory activity. Notably, at 0.1 mg/mL, PSBFE demonstrated inhibitory efficacy (79.1 ± 0.6%) comparable to the positive control drug acarbose (85.7 ± 0.6%), highlighting its potential as a natural α-glucosidase inhibitor (Table S4). The IC_50_ value of acarbose was 8.00 ± 0.01 μg/mL, while the IC_50_ values of SBFE and PSBFE were 131.04 ± 0.41 μg/mL and 52.89 ± 0.09 μg/mL, respectively (Table 4). Although PSBFE’s activity was lower than acarbose, it exhibited ~2-fold higher potency than the flavonoid-rich extract from *Clerodendrum glandulosum* Lindl (IC_50_ 104 μg/mL) [58]. The significant reduction in IC_50_ after purification (*p* < 0.05) underscores the enhanced efficacy of PSBFE, positioning it as a promising candidate for managing postprandial hyperglycemia through delayed carbohydrate digestion.

Consistent with previous findings, PSBFE exhibited stronger inhibitory activity against α-glucosidase than α-amylase [59]. This enhanced inhibition may be attributed to the abundant flavonoid glycosides present in PSBFE, which demonstrate superior α-glucosidase inhibitory effects compared to free flavonoids [60]. Quercetin, a natural polyphenol abundant in sea buckthorn pomace, has demonstrated significantly stronger α-glucosidase inhibition than acarbose in multiple studies, frequently serving as a positive control. Notably, its derivative isoquercetin exhibits even more potent activity, showing 32-fold greater inhibitory efficacy than the standard drug acarbose. The flavonoid extract also contains kaempferol and its glycosides, which similarly display potent α-glucosidase inhibition. Importantly, kaempferol’s selective inhibition profile—strong α-glucosidase suppression coupled with only mild α-amylase activity—suggests a potentially safer antidiabetic alternative to acarbose with reduced adverse effects [61]. To the best of our knowledge, this constitutes the first systematic investigation of the in vitro enzyme inhibitory potential of flavonoids derived from SBP. These findings collectively highlight the remarkable hypoglycemic potential of sea buckthorn pomace flavonoids. However, the precise molecular mechanisms underlying their antidiabetic effects warrant further investigation.

## 4. Conclusions

In this study, the flavonoids were efficiently extracted from sea buckthorn pomace by UEAE. Both enzymatic and ultrasound-assisted methods significantly enhanced the extraction efficiency. Single-factor experiments were combined with the response surface methodology to obtain the optimal extraction conditions: pectinase addition of 1500 U/g, ultrasonic power of 300 W, ethanol concentration of 48%, liquid-to-solid ratio of 34:1, extraction temperature of 50 °C, and extraction time of 28 min, achieved a flavonoid yield of 21.57 ± 0.92 mg/g. Among the factors investigated, the liquid–solid ratio demonstrated the greatest influence on extraction yield, followed by the ethanol concentration and extraction time. The main active components, kaempferol, quercetin, isorhamnetin, and their derivatives, may contribute to the SBP flavonoid extract’s robust antioxidant activity and significant hypoglycemic potential, since PSBFE can scavenge the free radical DPPH and ABTS, reduce ferric power, and inhibit α-amylase and α-glucosidase. These findings highlight SBP flavonoids as a promising natural resource for developing functional foods and nutraceuticals targeting diabetes management and oxidative stress-related disorders. Future research should focus on scaling up the extraction process, elucidating structure–activity relationships of the bioactive compounds, and evaluating their efficacy in in vivo models and clinical applications.

## Figures and Tables

**Table 1:** Box–Behnken design and results for response surface analysis.

Standard Order	Factors			Flavonoid Extraction Field (mg/g)
	(A) Extraction Time (min)	(B) Ethanol Concentration (%)	(C) Liquid–Solid Ratio (mL/g)	
1	15	40	30:1	21.08 ± 0.27
2	45	40	30:1	21.66 ± 0.12
3	15	80	30:1	18.58 ± 0.44
4	45	80	30:1	19.70 ± 0.04
5	15	60	20:1	17.63 ± 0.09
6	45	60	20:1	18.91 ± 0.25
7	15	60	40:1	20.54 ± 0.08
8	45	60	40:1	21.08 ± 0.12
9	30	40	20:1	19.02 ± 0.18
10	30	80	20:1	15.10 ± 0.32
11	30	40	40:1	20.77 ± 0.19
12	30	80	40:1	19.32 ± 0.21
13	30	60	30:1	21.45 ±0.28
14	30	60	30:1	21.71 ± 0.24
15	30	60	30:1	20.83 ± 0.20
16	30	60	30:1	20.67 ± 0.31
17	30	60	30:1	21.44 ± 0.14

**Table 2:** Flavonoid compounds identified in sea buckthorn pomace extracts by UPLC-QTOF-MS.

Identified Compounds	Molecular Formula	Retention Time (min)	Found at *m*/*z* ([M-H^−^])	MS/MS Fragments	Reference
(-)-Gallocatechin	C_15_H_14_O_7_	1.66	305.0662	137.0247, 179.0344	[45]
Procyanidin B2	C_30_H_26_O_12_	2.58	577.134	125.0248, 289.0727, 407.0783, 425.0890, 577.1375	[46]
*L*-Epicatechin	C_15_H_14_O_6_	3.02	289.0715	179.0354, 245.0812	[45]
Quercetin-3-*O*-rutinoside (Rutin)	C_27_H_30_O_16_	11.49	609.1456	301.0347	[47]
Quercetin-3-*O*-glucoside	C_21_H_20_O_12_	12.35	463.0879	301.0357, 463.0878	[48]
Kaempferol-3-*O*-rutinoside	C_27_H_30_O_15_	14.77	593.1506	285.0414	[45]
Isorhamnetin-3-*O*-neohespeidoside	C_28_H_32_O_16_	15.70	623.1611	315.0509	[49]
Myricetin	C_15_H_10_O_8_	18.00	317.03	137.0252, 151.0035, 178.9992	[44]
Kaempferol-3-*O*-glucoside	C_21_H_20_O_11_	19.44	447.0922	285.0422	[45]
Quercetin	C_15_H_10_O_7_	22.91	301.0353	151.0043, 178.9990	[48]
Apigenin-7-glucoside	C_21_H_20_O_10_	23.04	431.0975	151.0040, 257.0444	[49]
Naringenin	C_15_H_12_O_5_	25.84	271.0611	119.0512, 151.0034	[50]
Kaempferol	C_15_H_10_O_6_	27.18	285.0404		[48]
Isorhamnetin	C_16_H_12_O_7_	27.88	315.051	151.0038	[48]

**Table 3:** Antioxidant activity assay of different samples.

	FRAP (mmol FeSO_4_·7H_2_O/g)	DPPH (μmol Trolox/g)	ABTS (μmol Trolox/g)
SBFE	0.50 ± 0.01	208.58 ± 2.21	176.67 ± 0.51
PSBFE	2.68 ± 0.01	947.17 ± 3.85	427.33 ± 0.67
VC	13.50 ± 0.33	3050.00 ± 83.89	4271.11 ± 3.85

SBFE: sea buckthorn flavonoid extract; PSBFE: purified product sea buckthorn flavonoid extract; VC: vitamin C, positive control.

**Table 4:** α-Glucosidase and α-amylase inhibitory activities of different samples.

Types of Enzyme	Sample	IC_50_ (μg/mL)
α-glucosidase	SBFE	52.89 ± 0.09
	PSBFE	131.04 ± 0.41
	Acarbose	8.00 ± 0.01
α-amylase	SBFE	97.81 ± 0.42
	PSBFE	316.70 ± 1.43
	Acarbose	18.44 ± 0.08

SBFE: sea buckthorn flavonoid extract; PSBFE: purified product sea buckthorn flavonoid extract; Acarbose: positive control.

## Data Availability

The original contributions presented in this study are included in the article/Supplementary Material. Further inquiries can be directed to the corresponding authors.

## Supplementary Materials

The following supporting information can be downloaded at https://www.mdpi.com/article/10.3390/foods14101656/s1, Table S1: Independent variables and their levels used for Box-Behnken design. Table S2: Effect of different extraction method on flavonoid extraction yield from SBP. Table S3: Analysis of Variance (ANOVA) for response surface quadratic model. Table S4: α-Glucosidase and α-amylase inhibitory activities of different samples. Figure S1: Different extraction methods on total flavonoid yield. Figure S2: Chromatographic profile at 360 nm of purified sea buckthorn flavonoid extracts (PSBFE).
