# Supplementary material for: Optimization of Ultrasonic-Enzymatic-Assisted Extraction of Flavonoids from Sea Buckthorn (Hippophae rhamnoides L.) Pomace: Chemical Composition and Biological Activities
Source: Foods. 2025 May 8;14(10):1656. doi: 10.3390/foods14101656 (PMC12111566; doi:10.3390/foods14101656)
Supplement: Supplementary file 1 [file foods-14-01656-s001.zip › foods-3578872-supplementary.pdf]

Table S1. Independent variables and their levels used for Box-Behnken design.

Independent variables	Level		
	**-1**	**0**	**1**
Extraction time (A, min)	15	30	45
Ethanol concentration (B, %)	40	60	80
Liquid-solid ratio (C)	20:1	30:1	40:1

Table S2. Effect of different extraction method on flavonoid extraction yield from SBP.

Extraction conditions	Extraction yield (mg/g)
solvent extraction (SE)	9.90 ± 0.01
Ultrasonic-assisted extraction (UAE)	20.16 ± 0.02
Enzymatic-assisted extraction (EAE)	17.13 ± 0.17
Ultrasonic-enzymatic-assisted extraction (UEAE)	21.83 ± 0.01

Table S3. Analysis of Variance (ANOVA) for response surface quadratic model.

**Source**	**Sum of Squares**	***df***	**Mean Square**	***F*-value**	***p*-value**	
Model	47.63	9	5.29	36.96	< 0.0001	Significant
A	1.59	1	1.59	11.08	0.0126	*
B	12.16	1	12.1	84.94	< 0.0001	**
C	15.27	1	15.27	106.61	< 0.0001	**
AB	0.0745	1	0.0745	0.5205	0.4940	
AC	0.1388	1	0.1388	0.9691	0.3577	
BC	1.52	1	1.52	10.63	0.0138	*
A^2	0.0003	1	0.0003	0.0021	0.9644	
B^2	4.00	1	4.00	27.97	0.0011	**
C^2	12.01	1	12.01	83.88	< 0.0001	**
Residual	1.00	7	0.1432			
Lack of fit	0.1974	3	0.0658		0.8073	Not significant
Pure error	0.8049	4	0.2012			
Cor. total	48.63	16				
R^2	0.9794					
R^2_{Adj}	0.9529					
R^2_{pred}	0.9092					
CV_d	0.01					

[1]Analysis of Variance (ANOVA) for response surface quadratic model.

A- extraction time, *B*- ethanol concentration, *C*- liquid-solid ratio, *df*, Degrees of Freedom, CV_d, Coefficient of variation,

* Significant difference ($p < 0.05$).

** Highly significant difference ($p < 0.01$).

Table S4. α-Glucosidase and α-amylase inhibitory activities of different samples

Types of enzyme	Sample	Inhibition rate
α-glucosidase	SBFE	49.4 ± 0.1% (0.1mg/mL)
	PSBFE	79.1 ± 0.6% (0.1mg/mL)
	Acarbose	85.7 ± 0.6% (0.1mg/mL)
α-amylase	SBFE	15.9 ± 0.1% (0.3mg/mL)
	PSBFE	50.8 ± 0.1% (0.1mg/mL)
	Acarbose	67.8 ± 1.2% (0.03mg/mL)

[1]SBFE: sea buckthorn flavonoid extracts; PSBFE: purified product sea buckthorn flavonoid extracts; Acarbose: positive control.

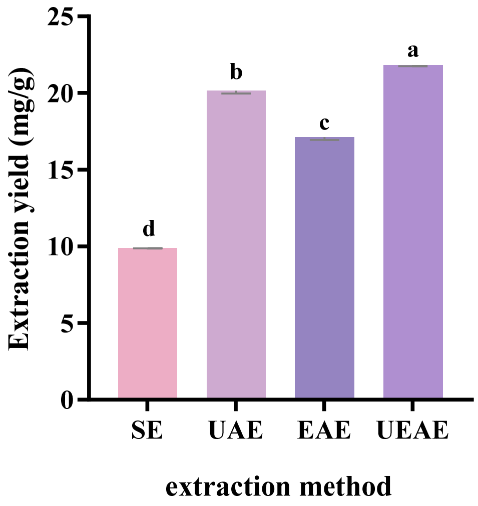

Figure S1. Different extraction methods on total flavonoid yield.
SE: solvent extraction; UAE: ultrasonic-assisted extraction; EAE: enzymatic-assisted extraction; UEAE: ultrasonic-enzymatic-assisted extraction.

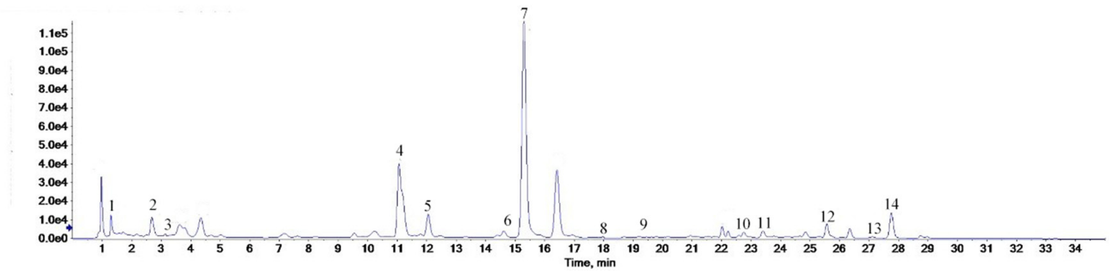

Figure S2. Chromatographic profile at 360 nm of purified sea buckthorn flavonoid extracts (PSBFE).

Peaks: (1), (-)-gallocatechin; (2), procyanidin B2; (3), *L*-epicatechin; (4), quercetin-3-*O*-rutinoside (rutin); (5), quercetin-3-*O*-glucoside; (6), kaempferol-3-*O*-rutinoside; (7), isorhamnetin-3-*O*-neohespeidoside; (8), myricetin; (9), kaempferol-3-*O*-glucoside; (10), quercetin; (11), apigenin-7-glucoside; (12), naringenin; (13), kaempferol; (14), Isorhamnetin.
